# Including RNA secondary structures improves accuracy and robustness in reconstruction of phylogenetic trees

**DOI:** 10.1186/1745-6150-5-4

**Published:** 2010-01-15

**Authors:** Alexander Keller, Frank Förster, Tobias Müller, Thomas Dandekar, Jörg Schultz, Matthias Wolf

**Affiliations:** 1 Department of Bioinformatics, University of Würzburg, Am Hubland, 97074 Würzburg, Germany

## Abstract

**Background:**

In several studies, secondary structures of ribosomal genes have been used to improve the quality of phylogenetic reconstructions. An extensive evaluation of the benefits of secondary structure, however, is lacking.

**Results:**

This is the first study to counter this deficiency. We inspected the accuracy and robustness of phylogenetics with individual secondary structures by simulation experiments for artificial tree topologies with up to 18 taxa and for divergency levels in the range of typical phylogenetic studies. We chose the internal transcribed spacer 2 of the ribosomal cistron as an exemplary marker region. Simulation integrated the coevolution process of sequences with secondary structures. Additionally, the phylogenetic power of marker size duplication was investigated and compared with sequence and sequence-structure reconstruction methods. The results clearly show that accuracy and robustness of Neighbor Joining trees are largely improved by structural information in contrast to sequence only data, whereas a doubled marker size only accounts for robustness.

**Conclusions:**

Individual secondary structures of ribosomal RNA sequences provide a valuable gain of information content that is useful for phylogenetics. Thus, the usage of ITS2 sequence together with secondary structure for taxonomic inferences is recommended. Other reconstruction methods as maximum likelihood, bayesian inference or maximum parsimony may equally profit from secondary structure inclusion.

**Reviewers:**

This article was reviewed by Shamil Sunyaev, Andrea Tanzer (nominated by Frank Eisenhaber) and Eugene V. Koonin.

**Open peer review:**

Reviewed by Shamil Sunyaev, Andrea Tanzer (nominated by Frank Eisenhaber) and Eugene V. Koonin. For the full reviews, please go to the Reviewers' comments section.

## Background

In the last decades, traditional morphological systematics has been augmented by novel molecular phylogenetics. One advantage of molecular data is the increased amount of parsimonious informative characters retained from genes that are usable for the inference of evolutionary relationships. This transition from few morphological features to abundant nucleotide or amino acid information has been a breakthrough for investigations of species relationships [1].

However, genetic data often inherits ambiguous information about phylogenetic relationships. Especially for very closely or distantly related taxa, certain parts of data sets may contradict each other or carry insufficient information. Phylogeneticists counter such problems e.g. by increase of the marker's size by inclusion of more nucleotides, thus increasing the amount of available data [2]. Moreover, different markers are combined, so that for example nuclear or mitochondrial genes are concatenated to increase the power of phylogenetic inferences [3,4]. These methods however face new problems. Increase of the number of nucleotides does not necessarily improve the accuracy of a tree reconstruction. Stochastically, only the robustness of the results is increased, if the complete elongated sequence evolved under the same evolutionary constraints [5]. The second method, marker concatenation, combines genes that result from different evolutionary processes and thus indeed include different evolutionary signals that may improve accuracy. However, they need to be investigated with marker-specific phylogenetic procedures as e.g. varying substitution models [6-8].

In this study we evaluate an alternative method applicable to ribosomal RNA (rRNA) genes that increases information content without addition of nucleotides. As non-coding RNA fragments of the genome, the rRNA gene is generally capable of folding into a secondary structure. In most cases, these structures are necessary for cell function and are thus evolutionarily conserved. Accordingly, structural information may be treated as a conserved marker. Secondary structures of ribosomal RNA therefore offer an additional source of information for tree reconstruction. In particular this is a major advantage in cases where secondary structures are very conserved, yet mutations of nucleotides occur frequently. This applies to the internal transcribed spacer 2 (ITS2) of the eukaryote ribosomal cistron [9,10]. Its secondary structure is evolutionarily maintained as it is of importance in ribogenesis. By contrast, the evolutionary rate of its sequence is relatively high and it is not present in the mature ribosome.

ITS2 sequences have been commonly used to infer phylogenies. Moreover, several studies already included secondary structures in their analyses either by morphometrical matrices or by sequence-structure alignments [11-16]. All these studies agree that the resulting reconstructions are improved by the secondary structures. However, no study has investigated and evaluated this benefit in detail. Evaluations of phylogenetic procedures are typically performed by two different means: the most commonly applied confidence measure in phylogenetics is non-parametric bootstrapping. Bootstrap support values are a measure of robustness of the tree and allow identification of trees or parts of trees that are not unambiguously supported by the data [17,18]. The second point of interest is accuracy measured by the distance between the real and the reconstructed tree. As the 'real' biological tree of life is not available, a switch to sequence simulations along 'real' artificial trees is necessary [19]. In this study we (1) simulate ITS2 sequences along evolutionary trees and (2) compare the results of tree reconstructions by sequence only data and combined sequence-structure data. Additionally, (3) the benefit of structural data is compared with that of sequence elongation. Furthermore, (4) a small biological example of plant phylogeny is presented in which reconstructions that either base on sequence-only or sequence-structure data are compared.

## Results

The overall calculation time took 80,000 processor hours on our 40 nodes network cluster. Each node comprised four Xeon 2.33 GHz cores. In total 448 GB RAM were used by the cluster.

**Figure 1:**
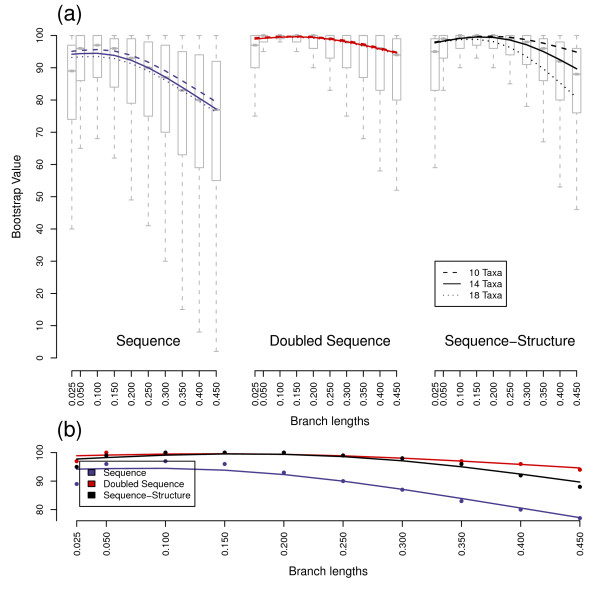
**Bootstrap support values for equidistant trees**. All five ancestral sequences were combined for a given scenario. (a) Boxplot and solid splines are for 14 taxa scenarios of the three methods. Dashed lines and dotted lines are splines of ten and 18 taxa, respectively. (b) Direct comparison of the 14 taxa splines and medians of all three methods. Sample sizes are 7,000, 11,000 and 15,000 for each of the ten, 14 and 18 taxa scenarios, respectively. Splines show a decrease of robustness with increased number of taxa used and increased branch lengths. Secondary structure and doubled sequences show an improvement in robustness in contrast to normal sequence information.

**Figure 2:**
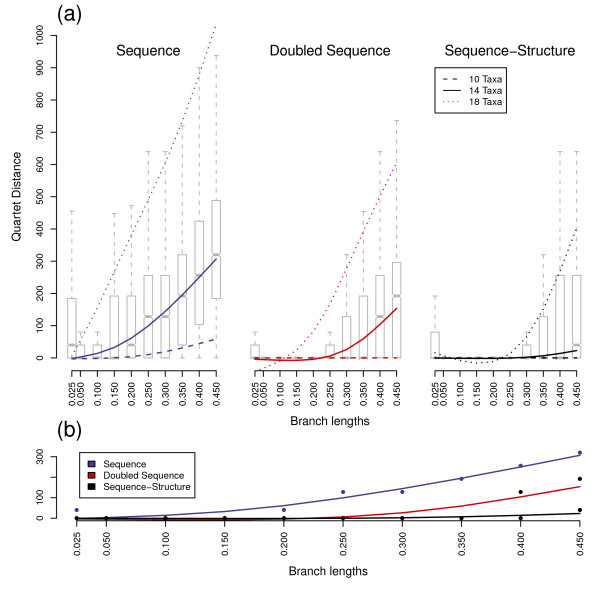
**Quartet distances values for equidistant trees**. All five ancestral sequences were combined for a given scenario. (a) Boxplot and solid splines are for 14 taxa scenarios of the three methods. Dashed lines and dotted lines are splines of ten and 18 taxa, respectively. (b) Direct comparison of the 14 taxa splines and medians of all three methods. The samples size of each scenario is 1,000. The accuracy of tree topologies decreases with more taxa and greater evolutionary distances between sequences. Trees calculated with secondary structures or doubled sequences show greater accuracy than those determined with normal sequences.

**Figure 3:**
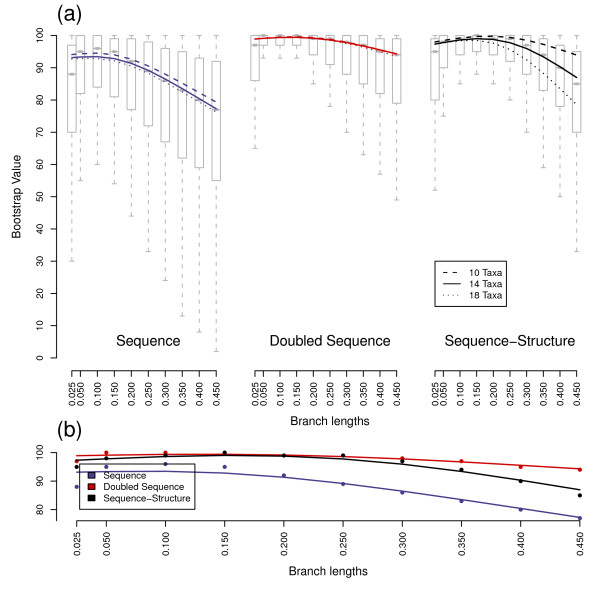
**Bootstrap support values for trees with variable branch lengths**. Subfigures are explained in Figure 1. Sample sizes are 7,000, 11,000 and 15,000 for each of the ten, 14 and 18 taxa scenarios, respectively.

**Figure 4:**
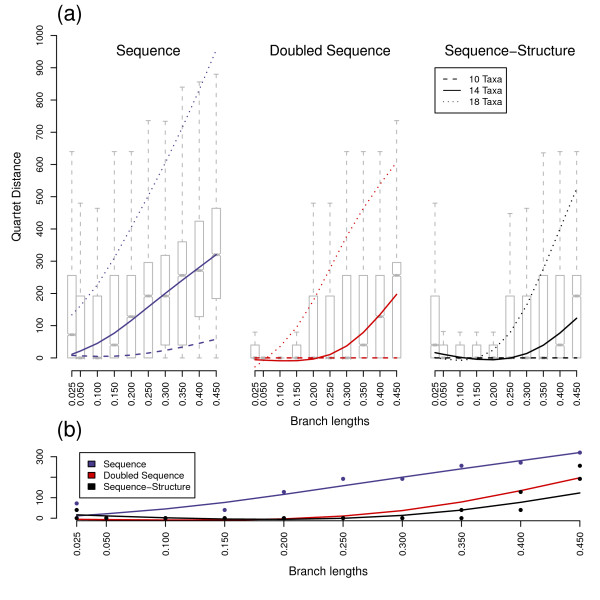
**Quartet distances values for trees with variable branch lengths**. Subfigures are explained in Figure 2. The samples size of each scenario is 1,000.

The shapes of bootstrap, Quartet distance and Robinson-Foulds distance distributions were similar for equidistant and variable distance trees. However, the branches of the trees for each underlying data set (sequence, sequence-structure and doubled sequence) received higher bootstrap support values and fewer false splits with constant branch lengths compared to variable distances, though differences were minimal (Figs. 1, 2, 3 and 4). Only Quartet distances are shown, since they are congruent with the results of the Robinson-Foulds distance (Additional file 1). Additionally, we included a relative per-branch representation of accuracy divided by the number of internal nodes in the Additional file 1.

Bootstrap values and tree distances obtained by differing ancestor sequences were similar in their distributions and thus combined for each scenario during the analysis process. Naturally, with increasing branch lengths, all three investigated data sets (sequences, doubled sequences and sequence-structure) became less accurate and robust, i.e. Quartet distances increased and bootstrap support of nodes decreased. This effect was also observable with an increasing number of external nodes.

Differences between the three methods also increased with evolutionary distance and number of taxa. Thus, the three methods (especially sequence-structure and doubled sequence) yielded almost similar results with low divergence (e.g. branch length 0.05) and few taxa (e.g. 10 taxa), whereas the results were different with branch lengths above 0.25 and at least 14 taxa.

For the lowest branch length we simulated, i.e. 0.025, in comparison to medium divergences a decreased accuracy and bootstrap support was observable with all three methods. This is explainable by too few base changes as providing information for phylogenetic tree reconstruction.

Sequence data performed best in reconstruction of trees (as the maximum and minimum of the spline-curves for bootstraps and tree distances, respectively) at a divergence level between 0.05 and 0.1. Sequence-structure shifted the optimal performance to higher divergences. This effect was also observable for doubled sequence, however it was not as prominent as for sequence-structure.

In general, the robustness of recalculated trees was highest for doubled sequence information contents. However, inclusion of secondary structures largely increased the bootstrap support values of nodes in contrast to normal sequence data. There is thus a robustness benefit to using secondary structure that is not directly comparable to benefits achieved by marker elongation.

Additionally, the accuracy of the trees benefitted from secondary structures: the number of false splits was significantly reduced compared to sequence as well as doubled sequence data. Thus sequences-structures yielded the most accurate results in our comparisons.

**Figure 5:**
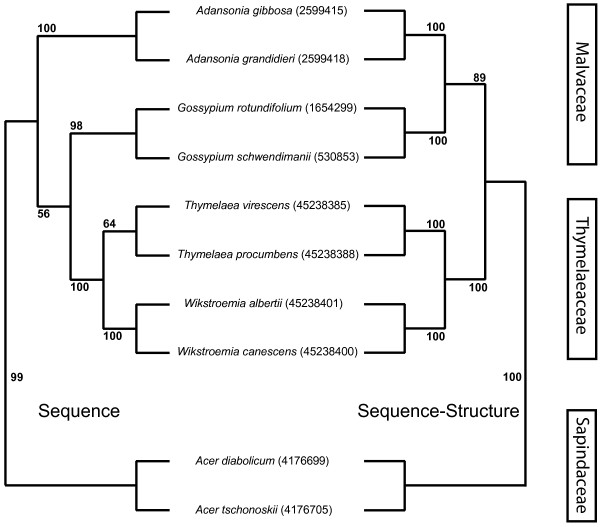
**Tree topology of the plants example**. Left side: topology and bootstrap values of sequence only data. Right side: corresponding tree with inclusion of secondary structure. Families of the species are given at the right end. GenBank identifiers are in parenthesis after the species names.

The results of trees reconstructed with sequence data and sequence-structure data for the plant example were very different. Sequence only information resulted in a correct topology reconstruction of genera (Fig. 5). However, the family of the Malvaceae could not be resolved. This supports the notion that the optimum divergence level of ITS2 sequences is at the species/genus level (see as well Additional file 2). By contrast, all genera and families could be resolved with secondary structures. This results in a flawless tree topology and highlights the improved accuracy. Furthermore, the robustness of the tree has been enhanced and the optimal divergence level has been widened.

## Discussion

### Number of Taxa and Divergence

Based on the simulations, we draw several conclusions regarding phylogenetic tree reconstructions with and without secondary structures. First of all, the robustness of a tree and its accuracy were significantly negatively correlated with number of taxa. This is the case even for normalized per-branch accuracy data (Additional file 1). Graybeal [20] argues that an increased taxon sampling enhances accuracy of a resolved tree in the 'Felsenstein zone'. We argue that such an enhancement is the case for special occurrences of long branch attraction, but not, according to our study, for general tree topologies. This is in accordance with Bremer et al. [2] as well as Rokas and Carroll [21], who also notice a slight decrease in accuracy with increased taxon sampling.

Secondly, according to Yang [22], a gene has an optimum level of sequence divergence for phylogenetic studies. The upper limits are reached when the observed difference is saturated, whereas the lower boundary is lack of information content caused by too few substitutions. We observed a similar pattern so that we are able to estimate the divergence level of best performance for ITS2 sequences with and without secondary structures. However, these differ for sequence data and sequence-structure data in two ways: inclusion of secondary structures shifted the best performance to a higher level of divergence. Thus, organisms that are more distantly related can be included in phylogenies. Furthermore, the range of optimal performance is wider for sequence-structure data. A shift to more distantly related sequences does not necessarily mean that relationships of closely related taxa are not any more resolvable. In a review Coleman [9] also identified this potential of ITS2 secondary structures by discussing several case studies. The small biological example of the Malvales and Sapindales in this study supports this notion. Our study mainly covers artificial data: a large scale comparison with biological data regarding the extension of the performance span is still desirable.

### Robustness and Accuracy

A substantial benefit to tree robustness was observable when including secondary structure information. Trees reconstructed with secondary structures are generally better bootstrap-supported by the data than those resulting from sequence only data [18]. This is caused by a gain of information content due to increased number of states possible for each nucleotide (unpaired, paired). This information is extractable with a suitable combined score matrix as implemented in 4SALE [23] or similar by site partitioning as in PHASE [24].

The major benefit we identified for phylogenetics is the improvement of accuracy. Sequences-structures performed far better than sequences alone in matching the 'real' tree, especially for high divergences. The resulting immense profit for phylogeneticists is obvious. It is the most crucial property of a phylogenetic tree to be as accurate as possible.

### Secondary structure vs. Marker elongation

Both, inclusion of secondary structures and increase of the number of nucleotides improved the reconstructed phylogenetic trees. However, inclusion of secondary structure in the reconstruction process is not equivalent to marker elongation. The major effect of more nucleotides is to increase the bootstrap support values. This has already been demonstrated by other authors [2,5]. With a theoretical increase of marker's length to infinitely large, corresponding bootstraps within a tree will stochastically be maximized as they exactly represent the data. In contrast, the benefit of secondary structures is predominantly the improvement of a tree's accuracy. Thus, additional sequence elongation and secondary structures represent different types of information increase. As the secondary structure analysis already covers the whole marker region of the ITS2 sequence, sequence elongation is not possible for real biological data.

The results retained in this study for the ITS2 region may be transfered to other ribosomal genes. However, the combination of a conserved secondary structure with a variable sequence seems to be of major benefit in phylogenetic studies. Other ribosomal markers, as e.g. 5.8S or 28S rRNA genes may profit less from addition of secondary structures than the ITS2, as the markers themselves are relatively conserved.

## Conclusions

Secondary structures of ribosomal RNA provide a valuable gain of information content that is useful for phylogenetics. Both, the robustness and accuracy of tree reconstructions are improved. Furthermore, this enlarges the optimal range of divergence levels for taxonomic inferences with ITS2 sequences. Thus, the usage of ITS2 sequence together with secondary structure for taxonomic inferences is recommended [25]. This pipeline is theoretically as well applicable to other reconstruction methods as maximum likelihood, bayesian inference or maximum parsimony. They may equally profit from secondary structure inclusion.

## Methods

### Simulation of ITS2 Sequences

**Figure 6:**
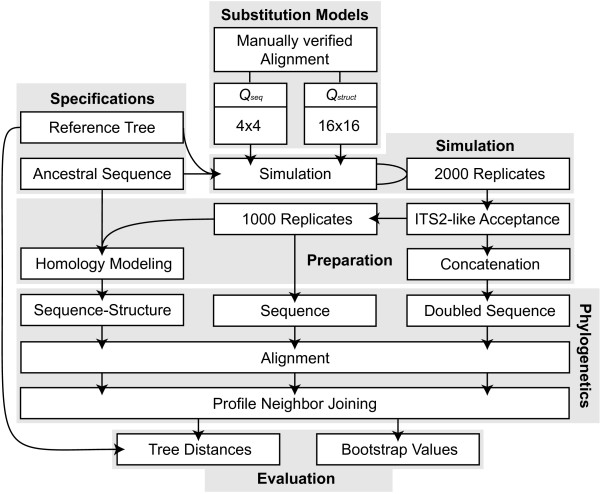
**Flowchart of simulation and phylogenetic reconstruction process**. Simulation of 2000 replicates of sequence sets was performed along the reference trees with an ancestral ITS2 sequence. Out of these, 1000 sequence-structure, sequence and concatenated sets were generated. Multiple sequence alignments were created for each of these sets and evolutionary distances were estimated with Profile Neighbor Joining. Resulting trees were afterwards compared with the reference trees and regarding their bootstrap support values. *Q*_*seq *_and *Q*_*struct *_are the substitution models for unpaired and paired regions, respectively.

Simulations of ITS2 sequences were performed with SISSI v0.98 [26]. Secondary structures were included in the simulation process of coevolution by application of two separate substitution models (Fig. 6, Additional file 3: Tab. 1 and Tab. 2): firstly we used a nucleotide 4 × 4 GTR substitution model *Q*_*seq *_for the evolution of unpaired nucleotides and secondly a dinucleotide 16 × 16 GTR substitution model *Q*_*struct *_for substitution of bases that form stem regions [11,27]. *Q*_*seq *_and *Q*_*struct *_were both estimated by a manually verified alignment based on 500 individual ITS2 sequences and structures with a variant of the method described by Müller and Vingron [28]. For lack of information about insertion and deletion events in the ITS2 region, such were not included into the simulations.

**Figure 7:**
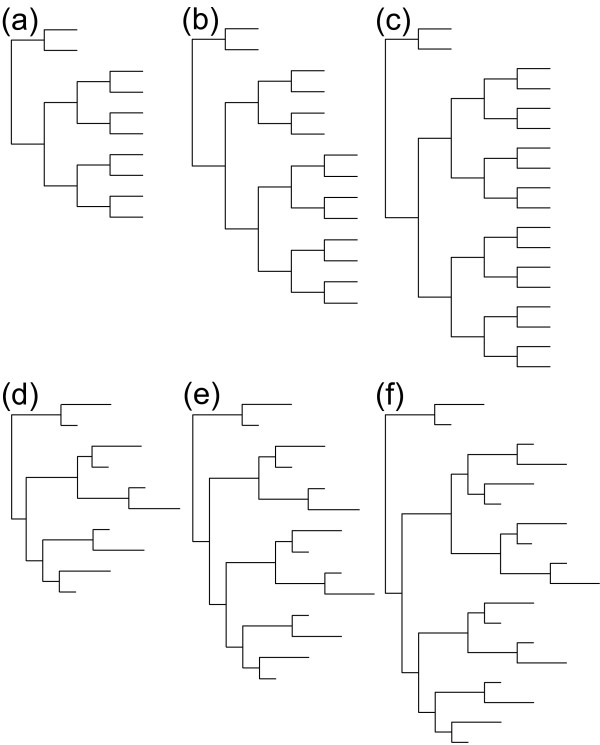
**Reference tree topologies used for simulation process**. Trees (a), (b) and (c) were trees with equidistance of branches. Trees (d), (e) and (f) were the corresponding variable trees with varying branch lengths. Trees (a) and (d) include ten taxa, (b) and (e) 14 taxa and (c) and (f) 18 taxa.

Simulations were started given (a) an ancestral sequence and (b) a reference tree that contained (c) specific branch lengths and (d) a certain number of taxa. In total, we used 10 different branch lengths, 5 ancestral sequences and 6 different trees (3 topologies for equal and variable branch length) resulting in 300 different combinatory conditions as evolutionary scenarios. (a) Ancestral sequences and structures were taken from the ITS2 database after HMM annotation [29-31]. They represented a cross section of the Eukaryota i.e. *Arabidopsis *(Plants) [GenBank:1245677], *Babesia *(Alveolata) [GenBank:119709754], *Gigaspora *(Fungi) [GenBank:3493494], *Gonium *(Green Algae) [GenBank:3192577] and *Haliotis *(Animals) [GenBank:15810877]. (b) The complete procedure was accomplished for two trees that shared a similar topology (Fig. 7). Tree shapes were chosen to resemble trees of a previously published simulation study [32]. The first was a tree that included constant branch lengths, whereas the second tree alternately varied +/- 50% of a given branch length. (c) The used branch lengths were 0.025, 0.05, 0.01, 0.15, 0.2, 0.25, 0.3, 0.35, 0.4 and 0.45. For comparison, pairwise distances of a typical phylogenetic study with ITS2 sequences have been added as Additional file 2. (d) Reference trees were calculated for 10, 14 and 18 taxa. The ancestral sequence served as an origin of the simulated sequences, but was not included in the reconstruction process and resulting tree.

Each simulated sequence set contained sequences according to the number of taxa. Sequence sets were accepted as composed of ITS2-like sequences if the structure of each sequence had been determinable by homology modeling with a threshold of 75% helix transfer [33]. For homology modeling, the ancestral sequence served as a template. Thus, each structure had four helices with the third helix as the longest. This acceptance scheme has been introduced for two reasons: the data is very similar to biological samples [10] and the structure prediction method is equal to that used at the ITS2 database [30] as well as phylogenetic reconstructions [25]. In total, 2,000 valid sequence sets were obtained for each scenario, what corresponds to 600,000 sequence sets summarized over all scenarios.

The complete sequence set is downloadable at the Supplements section of the ITS2 Database http://its2.bioapps.biozentrum.uni-wuerzburg.de/.

### Sequences and Structures of the Data Sets

Sequence data set: for each scenario, the order of the 2,000 simulated sequence sets retained from SISSI was shuffled. The first 1,000 were chosen and used as a sequence data set.

Sequence-structure data set: for each of the sequence sets used in the sequence data set, we determined the individual secondary structure of each sequence by homology modeling with at least 75% helix transfer [33]. The ancestral sequence was used as a template. Thus, for the sequence-structure data set we combined sequences with their respective secondary structures according to Seibel et al. [23]. Note, this approach using individual secondary structures is in contrast to alignments only guided by a consensus structure. Doubled nucleotide data set: The remaining 1,000 simulated sequence sets were used to exemplify effects on phylogenetic analyses of a hypothetical ITS2 gene size duplication. Each sequence of these sets was concatenated with a corresponding sequence of the sequence data set (same taxon in the simulation trees). Thus we received a data set of doubled nucleotide content that includes as well 1,000 sequence sets.

### Reconstruction of Simulated Phylogenetic Trees

For each simulated sequence set, ClustalW v2.0.10 [34] was used for calculation of multiple sequence alignments. In the cases of sequences and doubled sequences we used an ITS2 specific 4 × 4 scoring matrix [29,30]. For secondary structures, we translated sequence-structure information prior to alignment into pseudoproteins as described for 4SALE v1.5 [23,35]. Pseudoproteins were coded such that each of the four nucleotides may be present in three different states: unpaired, opening base-pair and closing base-pair. Thus, an ITS2 specific 12 × 12 scoring matrix was used for calculation of the alignment [23].

Reconstruction of phylogenetic trees for all trees has been performed with Profile Neighbor Joining (PNJ) of a console version of ProfDistS 0.9.8 [36,37]. With this we estimated improvements due to secondary structures, but keep the method of reconstruction constant. We decided in favor of PNJ and against other methods like maximum likelihood, Bayesian inference and parsimony for several reasons: the distance matrices are independent of insertion and deletion events, the algorithm is very fast and a pipeline for reconstructions with PNJ using secondary structures is already published [25]. However beneficial effects may be transferable to these methods. Profile building was allowed with default settings. General time reversible models (GTRs) were applied with the corresponding 4 × 4 and 12 × 12 substitution matrices for sequences and sequences-structures, respectively.

### Robustness and Accuracy

Profile Neighbor Joining trees were bootstrapped with 100 pseudo-replicates to retain information about the stability of the resulting tree. Bootstrap support values of all tree branches obtained from the 1,000 sequence sets of a certain scenario were extracted and pooled. Furthermore, the resulting trees were compared to the respective reference tree. In this regard, two tree distance quantification methods were applied, Robinson-Foulds distances using the Phylip Package v3.68 [38] and Quartet distances using Qdist v1.0.6 [39]. Results of all sequence sets were combined for a given scenario to receive the distributions of bootstrap values, Quartet distances and Robinson-Foulds distances, respectively. The result of each 14-taxa-scenario was plotted as a boxplot with notches using R v2.9.0 [40]. An interpolating spline curve was added. For the remaining scenarios (10 and 18 taxa) only spline curves were added for the sake of clarity.

### Short biological case study

Here we provide a short example of ITS2 secondary structure phylogeny, applied to biological data: we sampled sequences of three plant families using the ITS2-database browse feature (database accessed: June 2009): Thymelaeaceae (Malvales), Malvalceae (Malvales) and Sapindaceae (Sapindales). For each family we chose two sequences of the first two appearing genera. Tree reconstruction followed the methods described by Schultz and Wolf [25] and is equivalent to the reconstruction procedure used for the simulated sequence sets. Furthermore, the same procedure was applied without secondary structure information for comparison.

## Competing interests

The authors declare that they have no competing interests.

## Authors' contributions

AK, JS, MW and TD designed the study. FF and AK performed the simulation experiments and analyses. FF and TM estimated the substitution models used for simulations and reconstructions. AK, FF and MW drafted the manuscript. All authors contributed to writing the paper, read the final manuscript and approved it.

## Reviewers' comments

### Reviewer's report 1

Shamil Sunyaev, Division of Genetics, Dept. of Medicine, Brigham & Women's Hospital and Harvard Medical School

This manuscript demonstrates the utility of taking into account secondary structure in the phylogenetic analysis. Using comprehensive simulations and a real dataset of ITS2 sequences the authors demonstrated that for higher sequence divergence trees constructed with the help of secondary structure information improve accuracy and robustness. Another interesting result is that addition of taxa may reduce accuracy of tree reconstruction at least in terms of quartet distance between reconstructed and true trees.

#### Author's response

Thanks a lot for this positive report!

### Reviewer's report 2

Andrea Tanzer, Institute for Theoretical Chemistry, University of Vienna (nominated by Frank Eisenhaber, Bioinformatics Institute (BII) Agency for Science, Technology and Research, Singapore)

General comments:

The manuscript "Ribosomal Secondary Structures improve Accuracy and Robustness in Reconstruction of Phylogenetic Trees" compares different methods to improve the quality of phylogenetic analysis. RNA secondary structure information has been included in a variety of previous phylogenetic analysis, but this is the first study exploring the effect on the resulting trees in detail.

The authors use internal transcribed spacer 2 of ribosomal RNAs, a well established set of markers, to simulate a broad spectrum of 300 different scenarios. In addition, they compare their results from the simulations to a set of biological examples from selected plant species.

Overall, the manuscript is carefully written and the authors chose analysis and method appropriately. The simulated sequence set could be used for future studies.

Minor comments:

*) The title might be a little bit miss-leading since 'Ribosomal Secondary Structures' do not improve the 'Accuracy and Robustness in Reconstruction of Phylogenetic Trees' in general and the method should be applicable to other RNA markers. Therefore, I suggest something like "Including Secondary Structures improve Accuracy and Robustness in Reconstruction of Phylogenetic Trees".

*) The setup for the simulations is quite complex. It might help the reader if you add a table or figure to the supplemental material that summarizes the individual conditions for each data set produced.

Alternatively, you could just add to the text that you use 10 different branch length, 5 ancestral sequences and 6 different trees (3 topologies for equal and variable branch length) resulting in 300 different conditions. If I understand this correctly, then you retrieved for each of these 300 conditions 2,000 sequence sets (a total of 600,000 sets), where each set contains 10, 14 and 18 taxa, resp., depending on the tree topology used. These numbers should be mentioned in the text.

*) The set of simulated sequences should be accessible, such that it can be downloaded and used by the community for further studies. Maybe put a link on the website of the ITS2 database.

*) Predicting secondary structures of single sequences occasionally results in (mfe) structures of unexpected shapes. One way to get around this problem is the calculation of consensus structures of a set of related sequences. The resulting consensus structures can then be used for contraint folding of those sequences that could not be folded correctly in the first place. Furthermore, the sequences might fold into a number of equally good structures, but folding programs present only the first result (under default settings). The 'true' structure could as well be among the best folds, but not necessarily the optimal one (suboptimal folding). After all, folding algorithms only make the most plausible predictions. In this study, prediction of RNA secondary structures includes homology modelling. It is of question weather this is the most efficient method. However, since the structures deposited at the ITS2 database were created that way, it seems legitimate to apply it here a well.

#### Author's response

Thank you for carefully reading the manuscript. We addressed the minor comments regarding text changes and included the necessary information within the text. The set of simulated sequences is now downloadable at the Supplement section of the ITS2 Database http://its2.bioapps.biozentrum.uni-wuerzburg.de/. We totally agree that there are other possibly more efficient methods concerning structure prediction. However, as already stated by Dr. Tanzer 'structures deposited at the ITS2 database were created that way [homology modelling], it seems legitimate to apply it here as well'. The big advantage of the ITS2 is, that the core folding pattern is already known. Therefore, we have an external criterium to check for the correctness of the predicted structures.

### Reviewer's report 3

Eugene V. Koonin, National Center for Biotechnology Information, NIH, Bethesda

This is a useful method evaluation work that shows quite convincingly the inclusion of RNA secondary structure information into phylogenetic analysis improves the accuracy of neighbor-joining trees. My only regrets are about a certain lack of generality. It would be helpful to see a similar demonstration for for at least two different kinds of nucleic acid sequences not only ITS2. Also, at the end of the Conclusion section, the authors suggest that secondary structure could help also with other phylogenetic approaches (ML etc). Showing this explicitly would be helpful, especially, given that NJ is hardly the method of choice in today's phylogenetics.

#### Author's response

Thank you for your encouraging report. For ITS2 the core structure is well known and there are about 200,000 individual secondary structures available. However, it is absolutely right that it would be helpful to perform an analysis also on other types of phylogenetic RNA markers. Unfortunately, today there is no comparable amount of data available concerning secondary structures of other RNAs. Similarily, there are no programs to run an analysis on other methods such as parsimony, maximum likelihood and/or bayesian methods simultanously considering sequence and secondary structure information.

## Supplementary Material

Additional file 1 **Normalized Quartet distance and Robinson-Foulds plots**. Similar to Figures 2 and 4, but showing per-branch Quartet distances as a normalized standard i.e. divided by number of splits. Robinson-Foulds Distances are given in absolute and normalized versions. Click here for file

Additional file 2 **Empirical pairwise distances**. Pairwise distances of an ITS2 case study that integrates secondary structure. Click here for file

Additional file 3 **Substitution matrices**. Nucleotide 4 × 4 GTR substitution model *Q*_*seq *_for the evolution of unpaired nucleotides and a dinucleotide 16 × 16 GTR substitution model *Q*_*struct*_. Click here for file
