# Supplementary material for: Including RNA secondary structures improves accuracy and robustness in reconstruction of phylogenetic trees
Source: Biol Direct. 2010 Jan 15;5:4. doi: 10.1186/1745-6150-5-4 (PMC2821295; doi:10.1186/1745-6150-5-4)
Supplement: Additional file 1 — Normalized Quartet distance and Robinson-Foulds plots. Similar to Figures 2 and 4, but showing per-branch Quartet distances as a normalized standard i.e. divided by number of splits. Robinson-Foulds Distances are given in absolute and normalized versions. [file 1745-6150-5-4-S1.PDF]

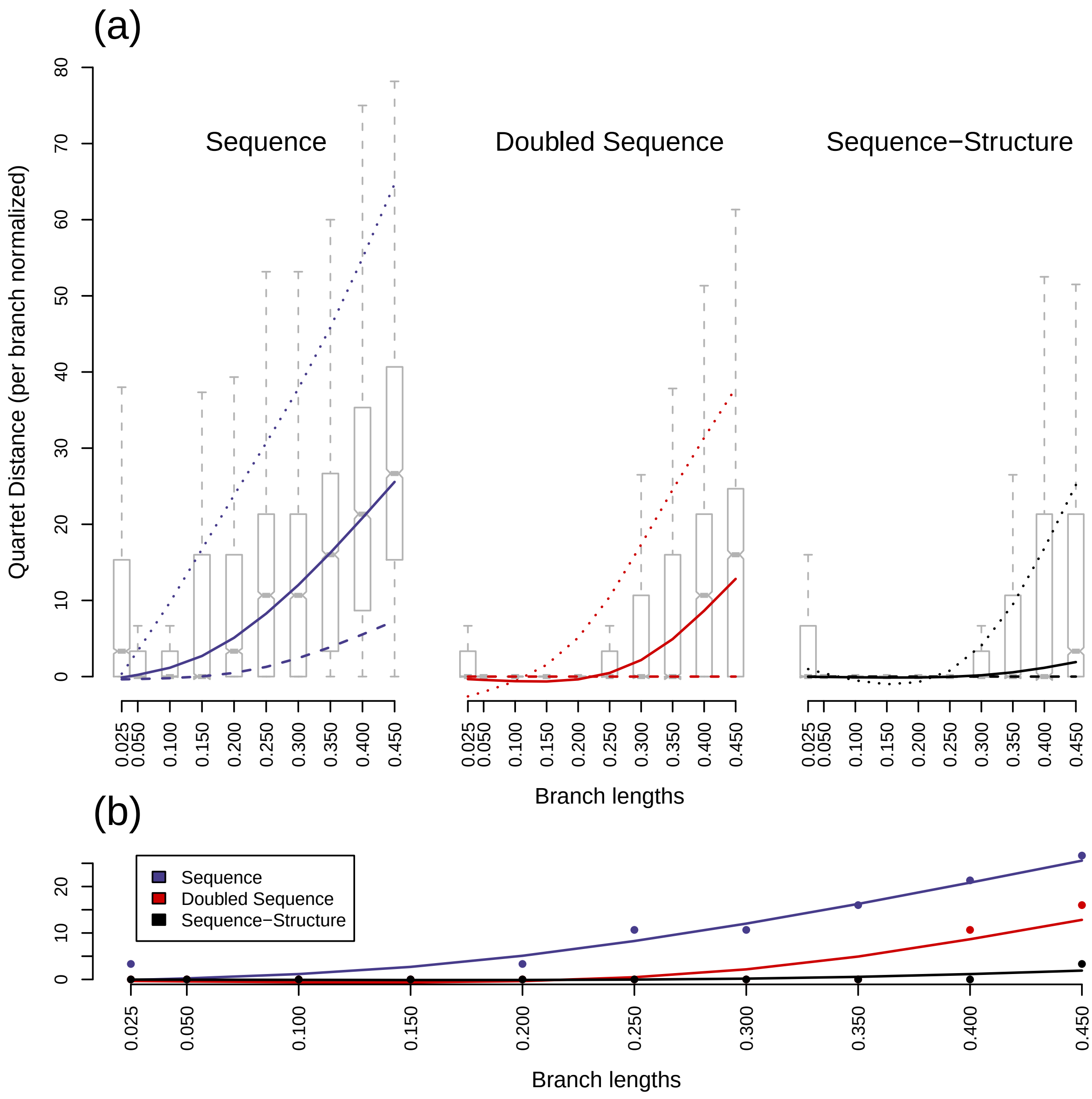

Additional file 1 Figure 1 - Normalized Quartet Distances for equidistant trees.

Similar to Figure 4, but showing per-branch Quartet distances as a normalized standard i.e. divided by number of splits.

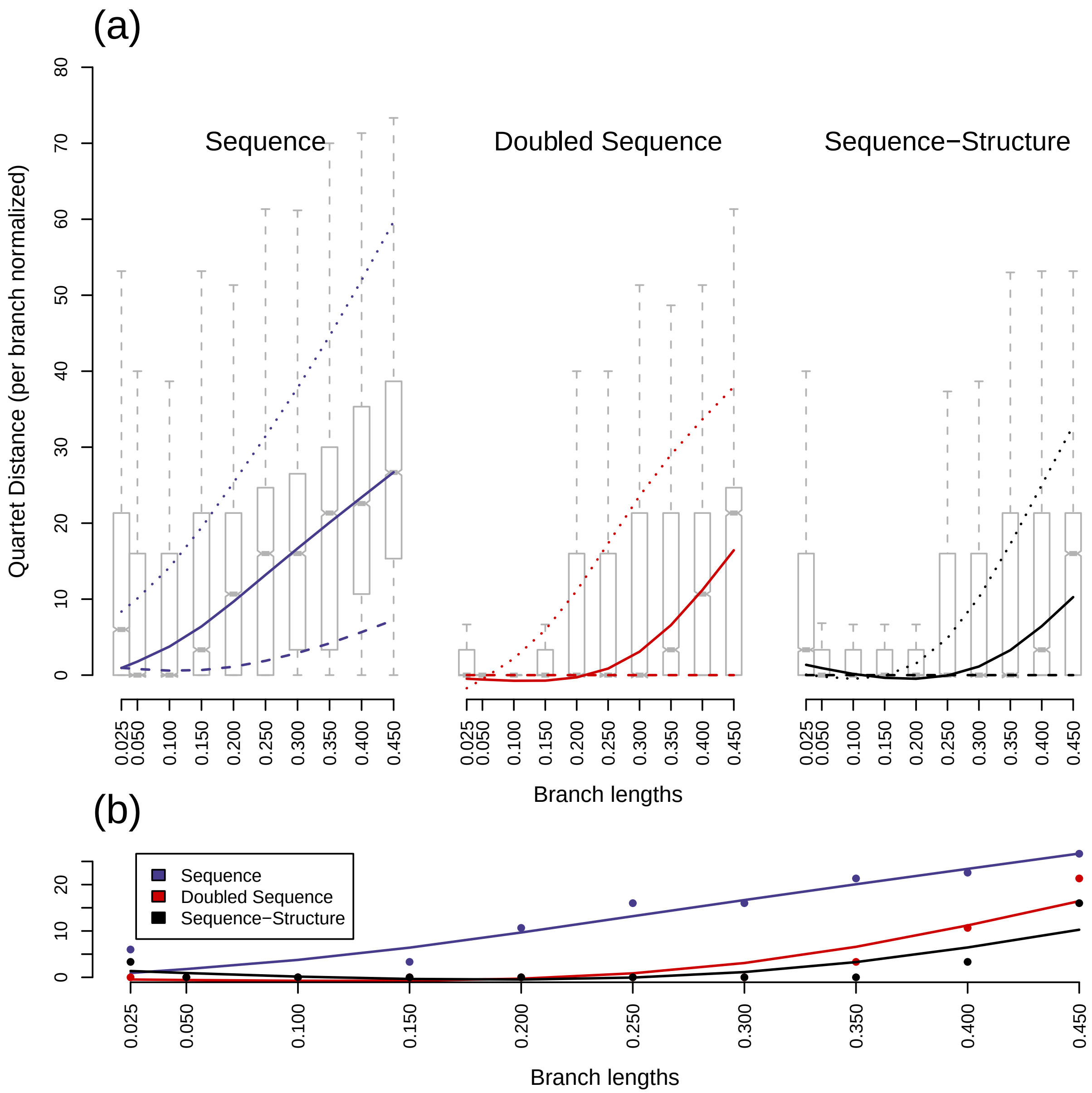

Additional file 1 Figure 2 - Normalized Quartet Distances for trees with variable branch lengths.

Similar to Figure 6, but showing per-branch Quartet distances as a normalized standard i.e. divided by number of splits.

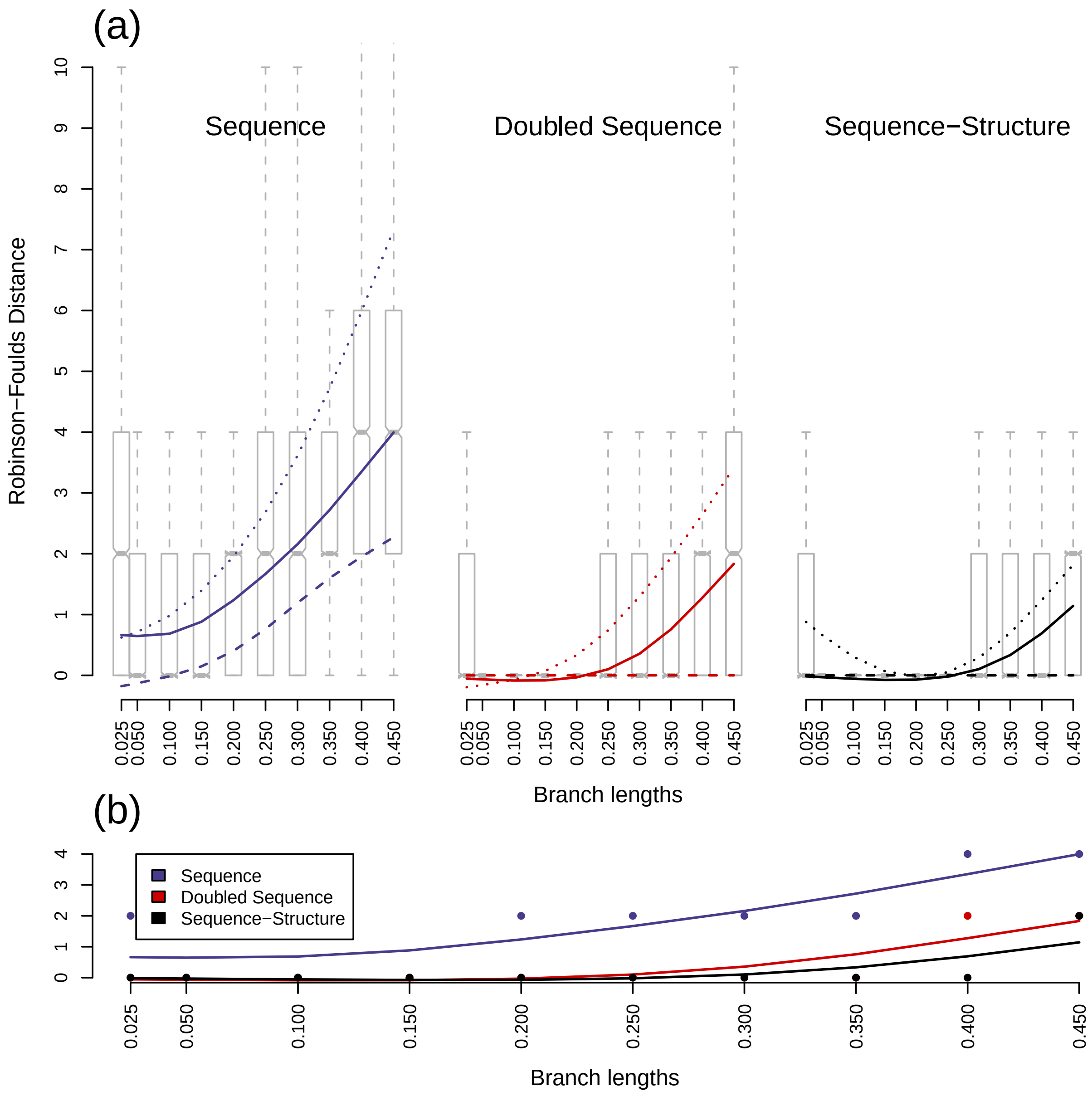

Additional file 1 Figure 3 - Absolute Robinson-Foulds Distances for equidistant trees.

Similar to Figure 4, but showing Robinson-Foulds distances.

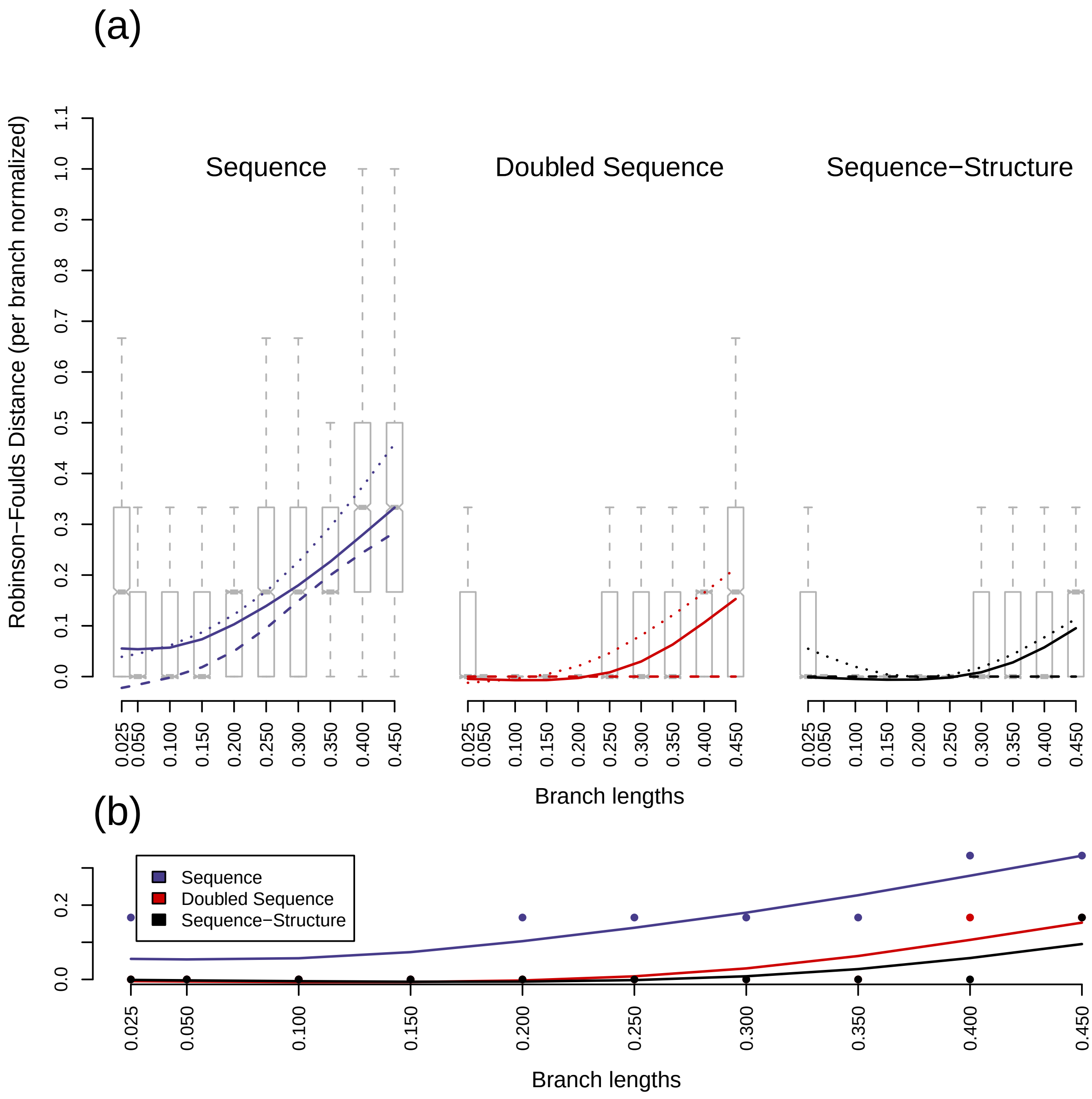

Additional file 1 Figure 4 - Normalized Robinson-Foulds Distances for equidistant trees.

Similar to Figure 4, but showing Robinson-Foulds distances as a normalized standard i.e. divided by number of splits.

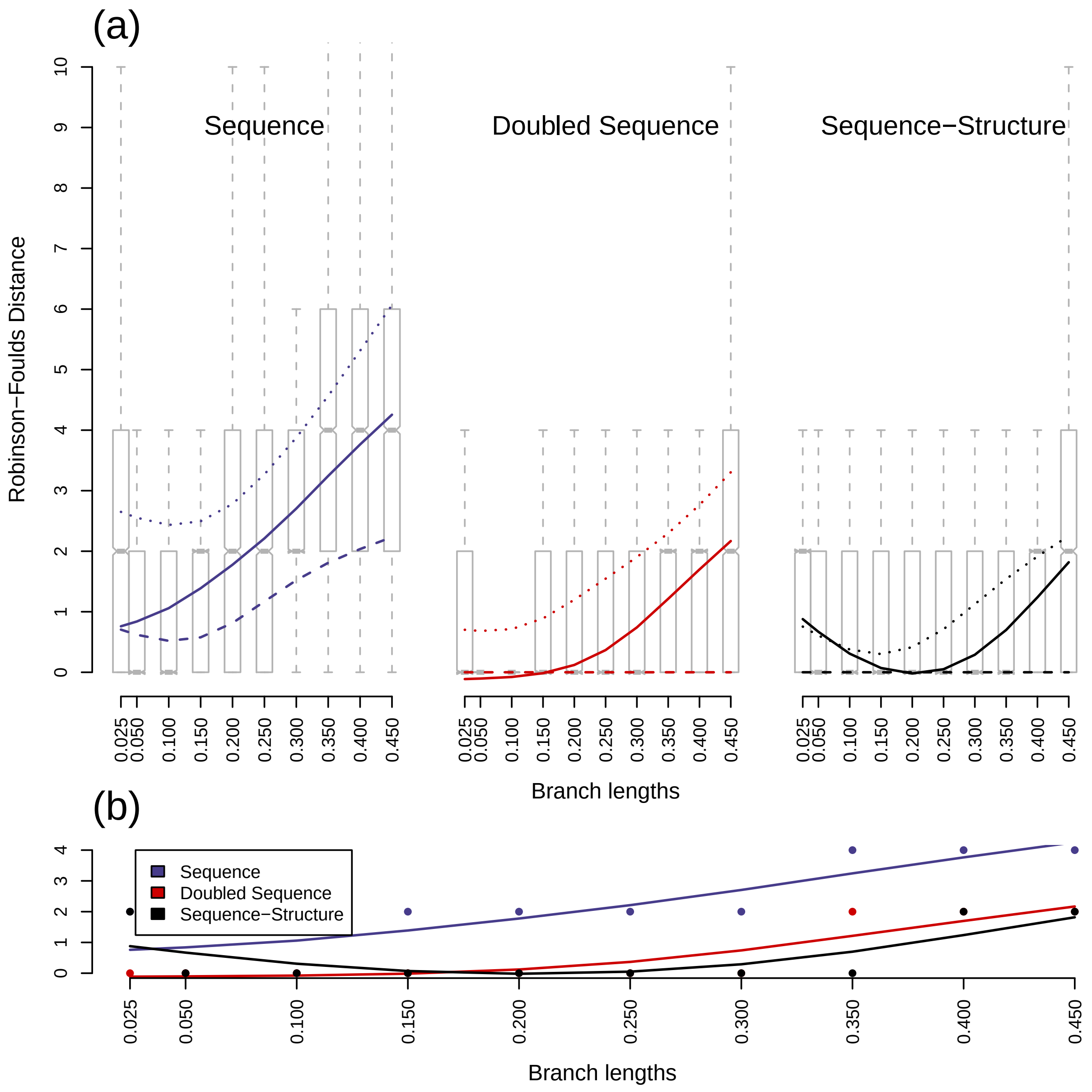

Additional file 1 Figure 5 - Absolute Robinson-Foulds Distances for trees with variable branch lengths.

Similar to Figure 6, but showing Robinson-Foulds.

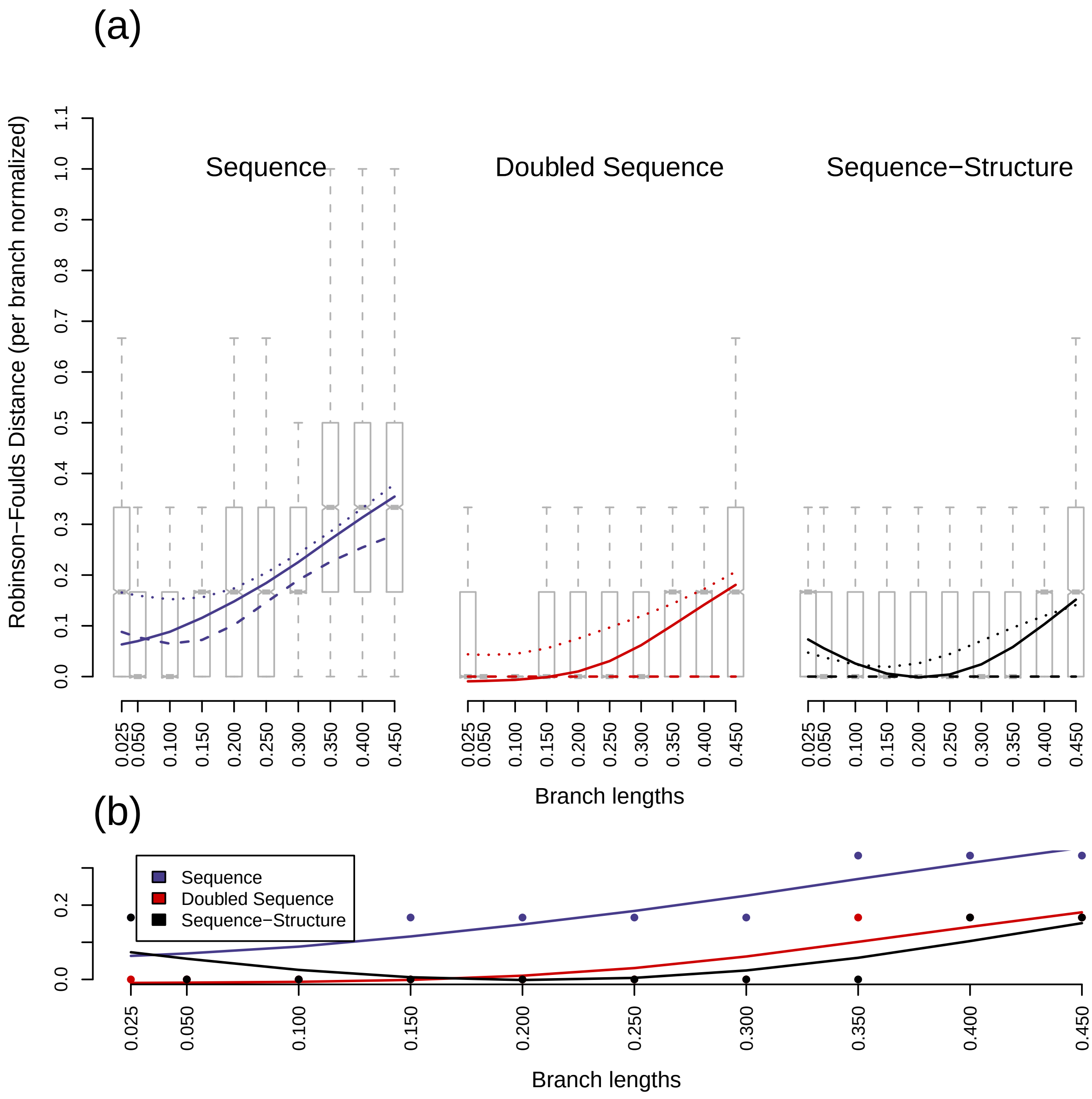

Additional file 1 Figure 6 - Absolute Robinson-Foulds Distances trees with variable branch lengths.

Similar to Figure 6, but showing Robinson-Foulds distances as a normalized standard i.e. divided by number of splits.
