# Supplementary material for: Including RNA secondary structures improves accuracy and robustness in reconstruction of phylogenetic trees
Source: Biol Direct. 2010 Jan 15;5:4. doi: 10.1186/1745-6150-5-4 (PMC2821295; doi:10.1186/1745-6150-5-4)
Supplement: Additional file 2 — Empirical pairwise distances. Pairwise distances of an ITS2 case study that integrates secondary structure. [file 1745-6150-5-4-S2.PDF]

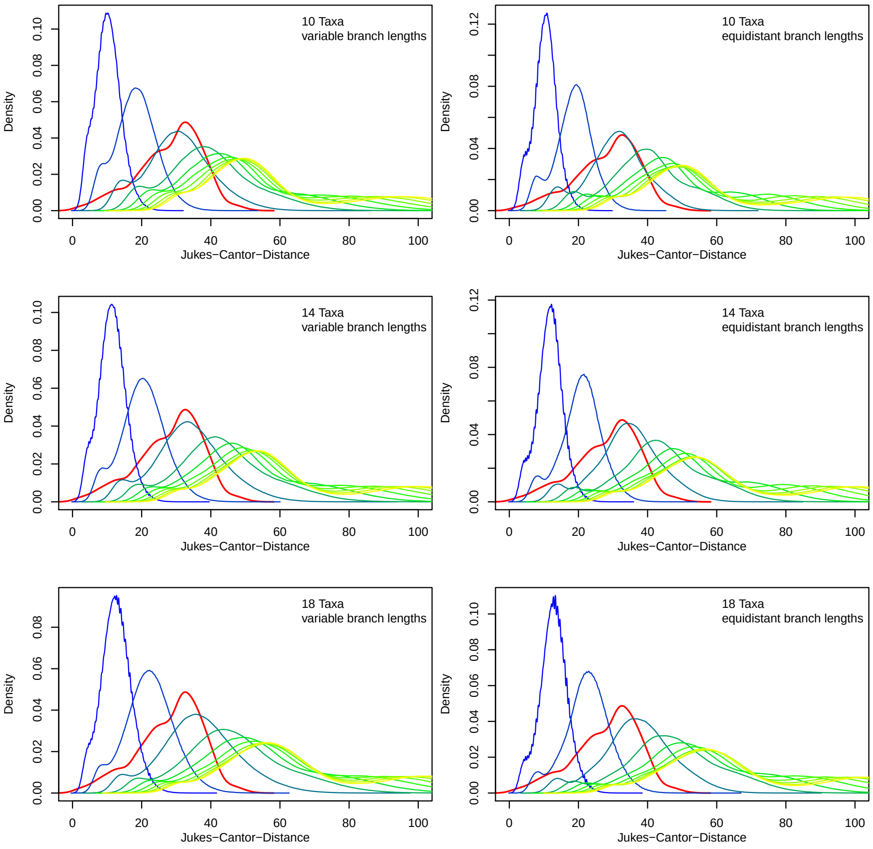

Additional file 2 Figure 1 - Jukes Cantor distances of simulated sequences and sequences of an empirical study.

Empirical sequences were taken from the study of Keller *et al.* [1]. Calculation of Jukes Cantor distance matrices per 100 basepairs was performed similarly for simulated and empirical sequences with Distmat of the EMBOSS package v.6.1.0 [2] after alignment with ClustalW v2.0.10 [3]. The density of the empirical distribution is shown in red, whereas simulated sequences are given as a color gradient from blue (branch length = 0.025) to yellow (branch length = 0.45).

References

1. Keller A, Schleicher T, Förster F, Ruderisch B, Dandekar T, Müller T, Wolf M: **ITS2 data corroborate a monophyletic chlorophycean DO-group (Sphaeropleales)**. *BMC Evol Biol* 2008, **8**:218.
2. Rice P, Longden I, Bleasby A: **EMBOSS: The European Molecular Biology Open Software Suite**. *TIG* 2000, **16**(6).
3. Thompson J, Higgins D, Gibson T: **ClustalW: improving the sensitivity of progressive multiple sequence alignment through sequence weighting, position-specific gap penalties and weight matrix choice**. *Nucleic Acids Res* 1994, **22**(22):4673–4680.
