# Supplementary material for: Including RNA secondary structures improves accuracy and robustness in reconstruction of phylogenetic trees
Source: Biol Direct. 2010 Jan 15;5:4. doi: 10.1186/1745-6150-5-4 (PMC2821295; doi:10.1186/1745-6150-5-4)
Supplement: Additional file 3 — Substitution matrices. Nucleotide 4 × 4 GTR substitution model Qseq for the evolution of unpaired nucleotides and a dinucleotide 16 × 16 GTR substitution model Qstruct. [file 1745-6150-5-4-S3.PDF]

Additional file 3 Table 1 - ITS2 speci c nucleotide relative rate matrix Q_{seq}

	A	C	G	U
A	0.000	0.945	2.297	1.117
C	0.945	0.000	1.040	2.973
G	2.297	1.040	0.000	1.000
U	1.117	2.973	1.000	0.000

Additional file 3 Table 2 - ITS2 speci c dinucleotide relative rate matrix Q_{struct}

	AA	AC	AG	AU	CA	CC	CG	CU	GA	GC	GG	GU	UA	UC	UG	UU
AA	0.000	0.000	0.000	0.000	0.039	0.000	0.000	0.000	0.522	0.000	0.000	0.000	1.056	0.000	0.000	0.000
AC	0.000	0.000	0.000	0.000	0.000	0.063	0.000	0.000	0.000	1.919	0.000	0.000	0.000	0.338	0.000	0.000
AG	0.000	0.000	0.000	0.000	0.000	0.000	1.023	0.000	0.000	0.000	0.327	0.000	0.000	0.000	1.839	0.000
AU	0.000	0.000	0.000	0.000	0.000	0.000	0.000	0.934	0.000	0.000	0.000	0.090	0.000	0.000	0.000	1.049
CA	0.039	0.000	0.000	0.000	0.000	0.000	0.000	0.000	0.005	0.000	0.000	0.000	1.082	0.000	0.000	0.000
CC	0.000	0.063	0.000	0.000	0.000	0.000	0.000	0.000	0.000	1.611	0.000	0.000	0.000	0.248	0.000	0.000
CG	0.000	0.000	1.023	0.000	0.000	0.000	0.000	0.000	0.000	0.000	1.042	0.000	0.000	0.000	0.155	0.000
CU	0.000	0.000	0.000	0.934	0.000	0.000	0.000	0.000	0.000	0.000	0.000	0.934	0.000	0.000	0.000	0.111
GA	0.522	0.000	0.000	0.000	0.005	0.000	0.000	0.000	0.000	0.000	0.000	0.000	1.061	0.000	0.000	0.000
GC	0.000	1.919	0.000	0.000	0.000	1.611	0.000	0.000	0.000	0.000	0.000	0.000	0.000	1.910	0.000	0.000
GG	0.000	0.000	0.327	0.000	0.000	0.000	1.042	0.000	0.000	0.000	0.000	0.000	0.000	0.000	1.735	0.000
GU	0.000	0.000	0.000	0.090	0.000	0.000	0.000	0.934	0.000	0.000	0.000	0.000	0.000	0.000	0.000	1.000
UA	1.056	0.000	0.000	0.000	1.082	0.000	0.000	0.000	1.061	0.000	0.000	0.000	0.000	0.000	0.000	0.000
UC	0.000	0.338	0.000	0.000	0.000	0.248	0.000	0.000	0.000	1.910	0.000	0.000	0.000	0.000	0.000	0.000
UG	0.000	0.000	1.839	0.000	0.000	0.000	0.155	0.000	0.000	0.000	1.735	0.000	0.000	0.000	0.000	0.000
UU	0.000	0.000	0.000	1.049	0.000	0.000	0.000	0.111	0.000	0.000	0.000	1.000	0.000	0.000	0.000	0.000
